# High Salt Intake Lowers Behavioral Inhibition

**DOI:** 10.3389/fnbeh.2019.00271

**Published:** 2019-12-13

**Authors:** T. Lee Gilman, Christina M. George, Mary Ann Andrade, Nathan C. Mitchell, Glenn M. Toney, Lynette C. Daws

**Affiliations:** ^1^Department of Cellular & Integrative Physiology, University of Texas Health Science Center at San Antonio, San Antonio, TX, United States; ^2^Addiction Research, Treatment & Training Center of Excellence, University of Texas Health Science Center at San Antonio, San Antonio, TX, United States; ^3^Center for Biomedical Neuroscience, University of Texas Health Science Center at San Antonio, San Antonio, TX, United States; ^4^Department of Pharmacology, University of Texas Health Science Center at San Antonio, San Antonio, TX, United States

**Keywords:** salt loading, stress, corticosterone, social behavior, anxiety, cardiovascular and neuropsychiatric comorbidity

## Abstract

Stress-related neuropsychiatric (e.g., anxiety, depression) and cardiovascular diseases are frequently comorbid, though discerning the directionality of their association has been challenging. One of the most controllable risk factors for cardiovascular disease is salt intake. Though high salt intake is implicated in neuropsychiatric diseases, its direct neurobehavioral effects have seldom been explored. We reported that elevated salt intake in mice augments neuroinflammation, particularly after an acute stressor. Here, we explored how high salt consumption affected behavioral responses of mice to mildly arousing environmental and social tests, then assessed levels of the stress-related hormone corticosterone. Unexpectedly, anxiety-related behaviors in the elevated plus maze, open field, and marble burying test were unaffected by increased salt intake. However, nest building was diminished in mice consuming high salt, and voluntary social interaction was elevated, suggesting reduced engagement in ethologically-relevant behaviors that promote survival by attenuating threat exposure. Moreover, we observed significant positive correlations between social preference and subsequent corticosterone only in mice consuming increased salt, as well as negative correlations between open arm exploration in the elevated plus maze and corticosterone selectively in mice in the highest salt condition. Thus, heightened salt consumption reduces behavioral inhibition under relatively low-threat conditions, and enhances circulating corticosterone proportional to specific behavioral shifts. Considering the adverse health consequences of high salt intake, combined with evidence that increased salt consumption impairs inhibition of context-inappropriate behaviors, we suggest that prolonged high salt intake likely promulgates maladaptive behavioral and cardiovascular responses to perceived stressors that may explain some of the prevalent comorbidity between cardiovascular and neuropsychiatric diseases.

## Introduction

Cardiovascular disease is closely associated with multiple neuropsychiatric diseases including post-traumatic stress disorder, depression, and anxiety (Player and Peterson, 2011; Cohen et al., 2015; Seldenrijk et al., 2015; Tully et al., 2016; Edmondson and Känel, 2017). Whether this is a unidirectional or bidirectional association remains unclear. One of the most controllable risk factors for cardiovascular disease is salt intake.

Among susceptible individuals, reducing dietary salt reduces cardiovascular disease risk, suggesting elevated salt consumption is capable of promoting cardiovascular disease (Aaron and Sanders, 2013; but see He and MacGregor, 2011; O’Donnell et al., 2013; Adler et al., 2014; Mozaffarian et al., 2014). Certainly, high salt intake is associated with numerous adverse health effects (Kotchen et al., 2013; Oh et al., 2016). High salt intake has also been touted as a suspected contributor to neuropsychiatric diseases (Abdoli, 2017). Indeed, higher sodium intake, indexed by elevated urinary sodium excretion, was recently associated with greater depressive symptom severity in adolescents (Mrug et al., 2019). Moreover, the combination of psychosocial stress – a key contributor to depressive and anxiety disorders – with increased salt intake has been recognized in humans as a potent precipitator of potentiated blood pressure, particularly in salt-sensitive individuals (Henry, 1988; Staessen et al., 1994; Deter et al., 1997, 2001). Such stress-salt interactions are suspected to contribute to the close association between stress-related neuropsychiatric diseases and cardiovascular disease (Mitchell et al., 2018; Gilman et al., 2019a).

Human dietary salt intake studies face challenges of controlling and verifying salt intake (O’Donnell et al., 2013; McLean, 2014), limiting our understanding of how salt intake levels in people impact responses to stress. Performing studies in mice, by contrast, makes controlling salt intake and exposure to specific stressors much more feasible, and allows for subsequent mechanistic investigations. Surprisingly though, few studies have examined how elevated salt intake in rodents influences subsequent *behavioral* responses to stressors, particularly social interactions. Instead, most studies to date have either focused on the impact of high salt intake on memory (Chugh et al., 2013; Ge et al., 2017), or the influence of high salt consumption plus social stress on blood pressure (Adams and Blizard, 1986). Recently, we have observed that elevated salt intake heightened active coping responses of mice to swim stress, and recruited a pro-inflammatory cytokine with associated activation of microglia and neurons in stress-responsive brain nuclei (Mitchell et al., 2018; Gilman et al., 2019a). Here, we sought to further assess how high salt consumption affects responses to relatively mild stressors by measuring anxiety-related and compulsive behaviors, as well as voluntary social interaction. We hypothesized that elevated salt intake would enhance anxiety-related and compulsive behaviors, and diminish social interaction.

## Materials and Methods

### Animals

**FIGURE 1:**
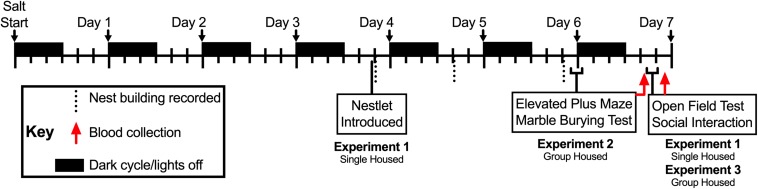
Timeline of Experiments 1, 2, and 3. Drinking manipulations commenced at lights off (1800 h; black rectangles) on Day 0 (i.e., Salt Start), and continued for up to 7 days. Mice in Experiments 2 and 3 were group housed, whereas mice in Experiment 1 were singly housed; the latter to enable individual measurements of nest building. Single nestlets were introduced into the cages of singly housed mice in Experiment 2 at 1315 h during the fourth day, and nest building was scored in the colony room at 1, 20, and 48 h time points thereafter (vertical dotted lines). Mice in Experiments 1 and 3 underwent open field testing (10 min) followed immediately by social interaction testing (10 min) between 1115 and 1415 h on the seventh day, then blood was collected 2 h after completion of social interaction testing (approximately 1340–1640 h; red arrow for Experiments 1 and 3). For Experiment 2, mice were tested in the elevated plus maze (5 min) between 1600 and 1800 h at the end of the sixth day, then immediately tested for marble burying (30 min). On the seventh day, 18 h after completion of the marble burying test, blood was collected from mice in Experiment 2 (approximately 1040–1310 h; red arrow for Experiment 2).

Male C57BL/6J mice purchased from Jackson Laboratories (Bar Harbor, ME, United States) were studied at ≥6 weeks of age. Once received, mice were group housed (2–5 mice per cage) in a temperature-controlled vivarium maintained at 24°C and on a 12:12 light:dark cycle, with lights on at 0600 h. All mice were housed on 7090 Teklad sani-chip bedding (Envigo, East Millstone, NJ, United States), in non-ventilated, open top cages, and given Teklad LM-485 mouse/rat sterilizable diet 7012 chow (Envigo) *ad libitum*. Mice were acclimated for at least 1 week prior to any salt intake manipulation; during this period they had *ad libitum* access to water. Once salt intake manipulations commenced, mice had *ad libitum* access only to the drinking solution to which their cage was assigned: water (0%), or 2 or 4% w/v sodium chloride (salt; Fisher Scientific, Waltham, MA, United States). Drinking solutions were introduced at lights off (1800 h) on the first day (indicated as “Salt Start” in Figure 1) and were accessible *ad libitum* for the duration of the experiment. At this time, mice either remained in their original group-housed cages (Experiments 2 and 3) or were singly housed (Experiment 1). No mice were in more than one Experiment. Access to each drinking solution was approximately 7 days for each experiment (see Figure 1; Mitchell et al., 2018; Gilman et al., 2019a). Mice used as social targets were age-matched male C57BL/6J mice bred in-house, group housed, and maintained under the same conditions as experimental mice, except all had *ad libitum* access to water and none were exposed to drinking solutions that contained salt. Though there is a disproportionate prevalence of emotional disorders in females compared to males, this is not as true of cardiovascular diseases prior to menopause (Maranon and Reckelhoff, 2013). Indeed, original salt loading models were established in males given the higher propensity of adults of this sex to suffer from cardiovascular diseases, and thus why we have begun our studies with this sex. Given that our previous demonstrations of psychogenic stressor response and neuroinflammation were in males (Mitchell et al., 2018; Gilman et al., 2019a), we sought to extend that work here, and examine females in future studies. All experiments were approved by the Institutional Animal Care and Use Committee at the University of Texas Health Science Center at San Antonio, and complied with the National Research Council’s Guide for the Care and Use of Laboratory Animals, 8th Ed.

### Behavior Testing

To reduce animal numbers, mice participated in at least two different behavior tests (see Figure 1; open field and social interaction, with or without nest building – Experiments 1 and 3, respectively; or elevated plus maze and marble burying – Experiment 2). Mice in Experiments 1 and 3 were submitted to open field and social interaction testing, both in an open field arena, after 6 days of drinking solution manipulation. Mice in Experiment 1 were singly housed from the onset of drinking solution manipulation to evaluate individual nest building behaviors beginning during the fourth day of drinking solution manipulation. Mice in Experiment 2 were tested in the elevated plus maze, followed immediately after by marble burying, on the sixth day of drinking solution manipulation. Blood collections took place 2 h after completion of social interaction testing (Experiments 1 and 3) or 18 h after completion of marble burying (Experiment 2). Nest building was scored in the colony room. For all other tests, mice were moved in their home cages from their colony room to the testing room at least 1 h before testing commenced, and were returned to their home cages and colony room at the end of testing.

#### Nest Building

To assess individual nest building behavior, mice were singly housed at the time of drinking manipulation onset. On the fourth day of drinking solution manipulation, a single nestlet (Ancare, Bellmore, NY, United States) was placed in each home cage at 1315 h in the colony room. Nest building was assessed in the colony room at 1, 20, and 48 h time points, and scores were assigned by a treatment-blinded observer as described by Deacon (2006).

#### Social Interaction Testing in Open Field Arena

An open field arena (42 cm wide × 42 cm long × 39 cm deep) was used for social interaction testing with slight modifications from previously described methods (Gilman et al., 2015; Latsko et al., 2016). Testing occurred between 1115 and 1415 h. Briefly, a wire cup (Spectrum Diversified Designs, LLC, Streetsboro, OH, United States) was placed inverted in one corner of the arena, and experimental mice were gently placed in the arena facing the corner opposite the inverted cup. Experimental mice were allowed to explore the arena and empty cup for 10 min (open field/target absent phase), then an age-matched male C57BL/6J social target was placed under the cup and experimental mice were allowed to explore for 10 more min (voluntary social interaction/target present phase). Sessions were recorded with a video camera for offline analysis with AnyMaze (Stoelting, Wood Dale, IL, United States). The center area was 20 cm square, and an entry required at least 80% of the animal’s entire body (tail excluded). Interaction with the empty or occupied cup was defined as at least 80% of the animal’s body being within a 7 cm wide arc of the cup. Social preference was calculated as the time spent interacting during the target present phase minus the time spent interacting during the target absent phase.

#### Elevated Plus Maze

Behavior in the elevated plus maze was assessed under dim white light conditions, between 1600 and 1800 h. The plus maze was constructed of white acrylic. Open arms measured 30 cm long × 5 cm wide, and closed arm walls were 15.5 cm high. The floor of the maze was elevated 51 cm above the floor, and all surfaces of the maze were cleaned with water between each animal tested. Elevated plus maze behaviors were video recorded for offline analysis with AnyMaze, in which 85% of the animal’s entire body (tail excluded) was required to be within an arm to qualify as an entry. Mice that fell off an open arm during the 5 min test were excluded from elevated plus maze analyses (0% salt: 1 of 9 excluded; 2% salt: 0 of 7 excluded; 4% salt: 1 of 10 excluded).

#### Marble Burying Test

Marble burying was evaluated as we previously described (Gould et al., 2011), and occurred under dim lighting between 1610 and 1840 h. Briefly, blue glass marbles were arranged in a 3 × 5 grid pattern atop 5–6 cm of wood chip bedding within a clear acrylic chamber (26 cm wide × 47 cm long × 20 cm high). Mice were then placed in the chamber and left undisturbed for 30 min, at which time pictures of the chambers were taken prior to removal of the mouse, to avoid any disturbance of the bedding. These images were used to quantify the number of marbles that were >25% visible; this number was subtracted from 15 to quantify how many marbles were >75% buried.

### Blood Collection and Processing

Trunk blood was collected under brief isoflurane anesthesia, either 2 h following completion of social interaction testing (Experiments 1 and 3) or 18 h after completion of marble burying (Experiment 2; Figure 1). Following previously published procedures (Latsko et al., 2016; Mitchell et al., 2018; Gilman et al., 2019b), blood was allowed to clot at room temperature for 1 h, then spun at 3500 rpm for 1 h at 4°C. The supernatant was then removed, and this serum was stored at −80°C until analysis.

### Corticosterone Analysis

Serum corticosterone levels were analyzed using an Enzo (Enzo Life Sciences, Inc., Farmingdale, NY, United States) corticosterone immunosorbent assay (cat. no ADI-900-097) as previously reported (Latsko et al., 2016; Mitchell et al., 2018; Gilman et al., 2019b).

### Serum Sodium

Serum Na^+^ concentrations were determined from duplicate samples using standard procedures with a PFP7 flame photometer (Techne, Minneapolis, MN, United States).

### Serum Osmolarity

Serum osmolarity was measured from duplicate samples using a model 3320 freezing point depression osmometer (Advanced Instruments, Norwood, MA, United States).

### Serum Protein

Similar to our previous description of analyzing plasma protein (Gilman et al., 2019b), quantification of serum protein involved pipetting 50 μL of serum onto a calibrated, hand-held manual refractometer (AtagoUSA, Inc., Bellevue, WA, United States) to determine specific gravity (g/100 ml).

### Statistical Analysis

Data from mice in Experiments 1 and 3 were analyzed with two-way ANOVAs (salt × housing conditions or salt × time for nest building). Corticosterone levels from mice in these two experiments were found by Shapiro–Wilk normality tests to be non-normally distributed, so for consistency and comparability, all corticosterone data were log-transformed using GraphPad Prism (v7.0e; GraphPad Software, La Jolla, CA, United States). Experiment 2 data, where mice were all group housed, were analyzed with one-way ANOVAs (salt condition). To minimize Type I error, *post hoc* tests were selected based upon the outcome of ANOVAs. When an interaction between salt × housing conditions was detected, Tukey’s *post hoc* testing was employed; Sidak’s *post hoc* was used when a main effect of housing was identified; Dunnett’s *post hoc* was implemented when a main effect of salt condition or a salt × time interaction was observed. Associations between behavior data and log-transformed corticosterone were analyzed with Pearson correlation analyses in GraphPad Prism. Significance was set *a priori* at *p* < 0.05, and data were graphed as individual data points (except for Figure 3B), with the mean indicated as a horizontal line (symbols represent mean for Figure 3B), and 95% confidence interval (CI) indicated with a vertical line.

## Results

### Elevated Plus Maze

**FIGURE 2:**
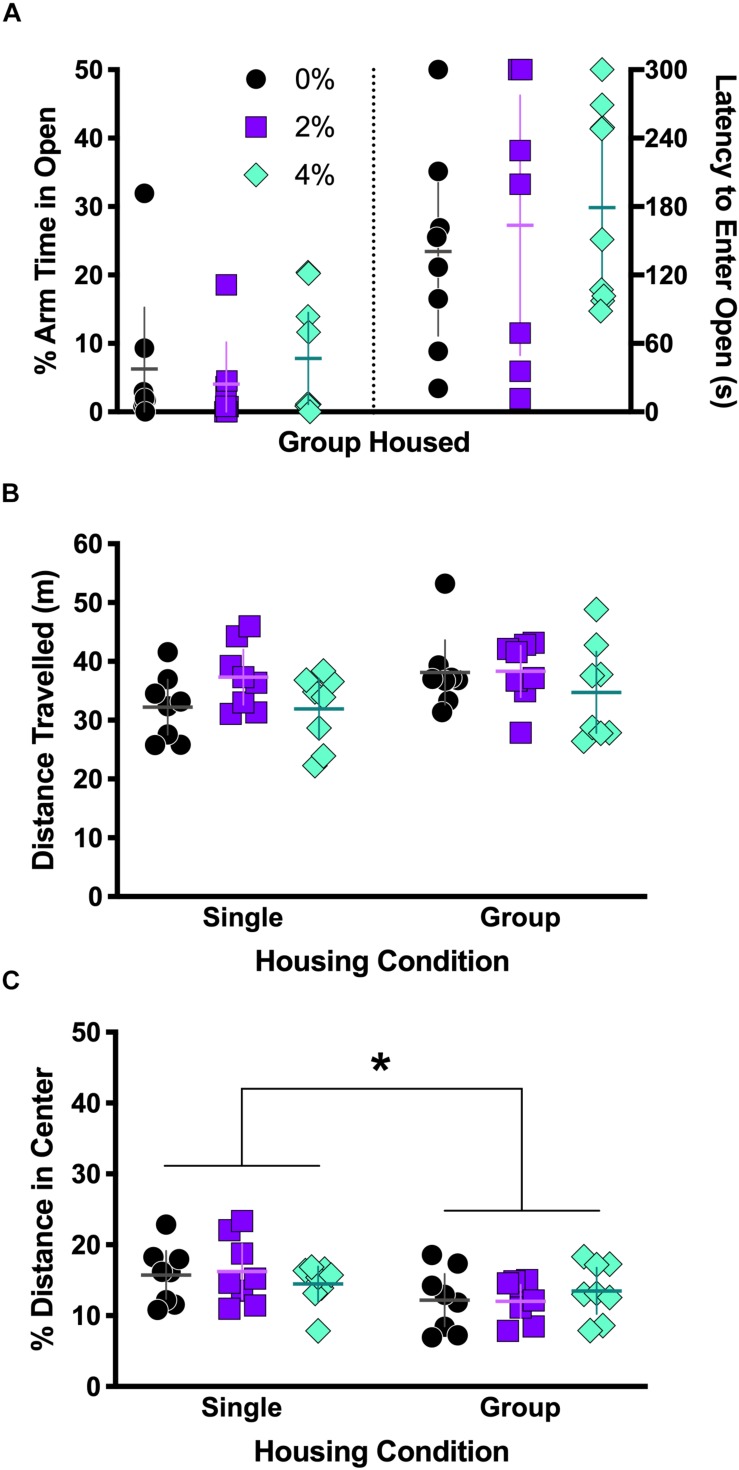
Behavior in elevated plus maze and open field test. Group housed mice from Experiment 2 were tested in the elevated plus maze (**A**). No differences were observed the percent of arm time spent in the open arm (**A**, left half) or latency to first entry in the open arm (**A**, right half). Single and group housed mice in Experiments 1 and 3, respectively, were tested in the open field (**B**,**C**). Neither salt nor housing condition significantly affected overall locomotor activity, measured as distance traveled (**B**), though a non-significant trend for a main effect of housing was noted (*p* = 0.085). There was a significant main effect of housing on the percent of total distance traveled in the center (**C**, ^∗^*p* = 0.013). Individual data points are shown, with means indicated by horizontal lines, and 95% CI indicated with vertical lines.

No significant differences in percent of arm time spent in the open arms [*F*(2,21) = 0.35, *p* = 0.71] or latency to enter the open arms [*F*(2,21) = 0.32, *p* = 0.72] were observed as a consequence of salt condition in group housed mice (Figure 2A).

### Open Field Test

Though locomotor activity was not affected by an interaction between housing and salt conditions [*F*(2,42) = 0.62, *p* = 0.54; Figure 2B], there was a non-significant trend noted for housing [*F*(1,42) = 3.1, *p* = 0.085]. No main effect of salt was observed on distance traveled [*F*(2,42) = 2.0, *p* = 0.14]. A significant main effect of housing on percent of distance traveled in the center area was detected [*F*(1,42) = 6.8, *p* = 0.013; Figure 2C] where single housed mice traveled a greater percent distance in the center area, but a Sidak’s *post hoc* test did not indicate any significant differences across housing condition within each salt condition. The interaction of housing and salt conditions [*F*(2,42) = 0.78, *p* = 0.46] was not significant, nor was salt condition alone [*F*(2,42) = 0.009, *p* = 0.99].

### Marble Burying Test

**FIGURE 3:**
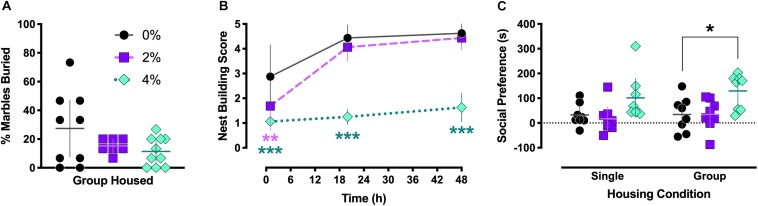
Marble burying, nest building, and social behaviors. Group housed Experiment 2 mice exhibited no significant differences in marble burying behavior (**A**). Nest building behavior was scored for singly housed mice in Experiment 1 at 1, 20, and 48 h after introduction of a nestlet (**B**). After 1 h, nest building was significantly impaired in both 2% (^∗∗^*p* = 0.0087) and 4% (^∗∗∗^*p* < 0.0001) salt-consuming mice. Nest building by mice consuming 4% salt remained inhibited at the 20 (^∗∗∗^*p* < 0.0001) and 48 h (^∗∗∗^*p* < 0.0001) time points. Preference for voluntary social interaction (**C**) was significantly increased in group housed mice in Experiment 3 (^∗^*p* = 0.015), and a non-significant trend for increased social interaction in singly housed Experiment 1 mice was noted (*p* = 0.086). For **(A,C)**, individual data points are shown, with means indicated by horizontal lines, and 95% CI indicated with vertical lines. Means (*N* = 8) are indicated with symbols in **(B)**, and vertical lines indicate 95% CI.

No significant differences in marble burying were observed in group housed mice [*F*(2,23) = 2.35, *p* = 0.12; Figure 3A].

### Nest Building

A significant interaction between salt condition and time was detected in single housed mice [*F*(4,42) = 7.0, *p* = 0.0002; Figure 3B]. Dunnett’s *post hoc* tests indicated that 1 h after introduction of the nestlet, animals in the 2% (*p* = 0.0087) and 4% (*p* < 0.0001) salt conditions had lower nest building scores than control 0% mice. This reduced nest building compared to controls persisted in mice in the 4% salt condition at 20 (*p* < 0.0001) and 48 h (*p* < 0.0001) time points.

### Social Interaction

Salt condition had a significant main effect on social interaction behavior [*F*(2,42) = 8.6, *p* = 0.0007; Figure 3C], but no significant effect of housing [*F*(1,42) = 0.65, *p* = 0.42] or interaction between housing and salt conditions [*F*(2,42) = 0.14, *p* = 0.87] was found. Dunnett’s *post hoc* testing revealed that, in the group housed condition, mice consuming 4% salt exhibited significantly greater social preference compared to control mice (*p* = 0.015). This comparison in single housed animals trended toward significance (*p* = 0.086).

### Serum Measures

**FIGURE 4:**
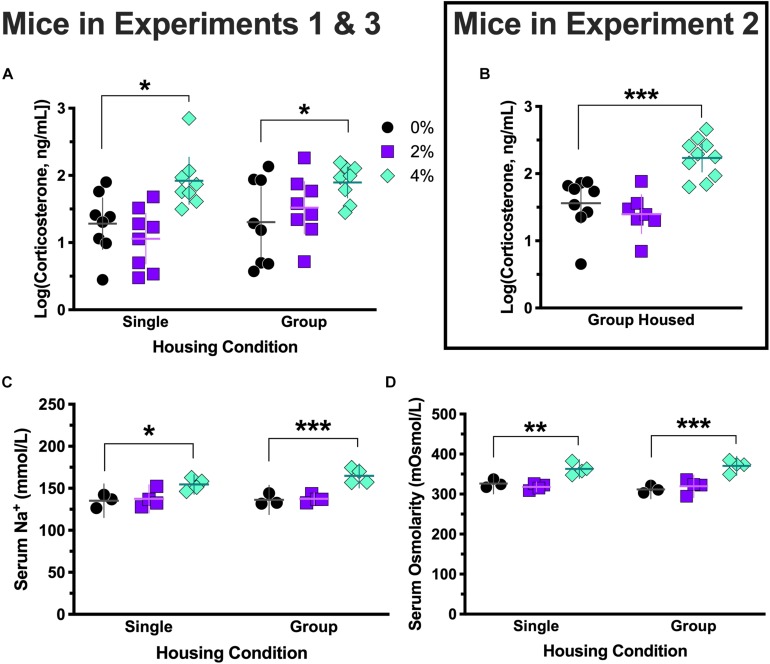
Log-transformed serum corticosterone levels, serum sodium levels, and serum osmolarity. Corticosterone levels in Experiment 3 (group housed) mice consuming 0 and 2% salt, and in Experiment 1 (singly housed) mice consuming 4% salt, were non-normally distributed, so all corticosterone levels were log-transformed to generate normally distributed data for ANOVA analyses. Log-transformed corticosterone levels consistently revealed elevated corticosterone in mice consuming 4% salt in Experiment 3 [group housed, ^∗^*p* = 0.026, **(A)** right side], Experiment 1 [single housed, ^∗^*p* = 0.016, **(A)** left side], and Experiment 2 [group housed, ^∗∗∗^*p* = 0.0004 (**B**)]. Serum sodium (Na^+^) levels were significantly higher in mice consuming 4% salt in both Experiment 3 [group housed, ^∗∗∗^*p* < 0.0005, **(C)** right side] and Experiment 1 [singly housed, ^∗^*p* = 0.011, **(C)** left side]. Serum osmolarity was also significantly elevated in both group [Experiment 3; ^∗∗∗^*p* < 0.0001, **(D)** right side] and single [Experiment 1; ^∗∗^*p* = 0.004, **(D)** left side] housed mice consuming 4% salt relative to control mice, as indicated by Dunnett’s *post hoc* testing. Individual data points are shown, with means indicated by horizontal lines, and 95% CI indicated with vertical lines.

Serum corticosterone levels in the group housed 0% (*W* = 0.80, *p* = 0.028), group housed 2% (*W* = 0.77, *p* = 0.015), and single housed 4% (*W* = 0.53, *p* < 0.0001) conditions in Experiments 1 and 3 failed to pass Shapiro–Wilk normality tests, so all corticosterone levels were log-transformed to generate normally distributed data for ANOVA analyses. No effect of housing [*F*(1,42) = 1.3, *p* = 0.26], nor an interaction between housing and salt conditions [*F*(2,42) = 1.4, *p* = 0.27], was detected (Figure 4A). However, a significant main effect of salt condition was observed [*F*(2,42) = 9.5, *p* = 0.0004]. Dunnett’s *post hoc* testing indicated that animals in the 4% salt condition had significantly elevated serum corticosterone levels relative to 0% controls in both the group (*p* = 0.026) and single (*p* = 0.016) housing conditions. Likewise, corticosterone levels from mice in Experiment 2 showed a significant effect of salt condition [*F*(2,23) = 15.8, *p* < 0.0001; Figure 4B], with *post hoc* testing revealing that the 4% salt condition had significantly higher serum corticosterone than controls (*p* = 0.0004).

Serum levels of sodium (Na^+^) were measured in subsets (*N* = 3–4) of mice from Experiments 1 and 3. Serum Na^+^ levels did not exhibit an interaction between housing and salt conditions [*F*(2,16) = 0.92, *p* = 0.42; Figure 4C]. No effect of housing was observed [*F*(1,16) = 1.2, *p* = 0.30], but a main effect of salt condition was significant [*F*(2,16) = 21.1, *p* < 0.0001]. *Post hoc* testing indicated that in both group (*p* < 0.0005) and single (*p* = 0.011) housing conditions, 4% salt mice had significantly greater serum Na^+^ than controls.

As with Na^+^ levels, serum osmolarity did not show a significant interaction between housing and salt conditions [*F*(2,16) = 1.2, *p* = 0.32], nor a main effect of housing [*F*(1,16) = 0.10, *p* = 0.75]. Salt condition did indicate a main effect [*F*(2,16) = 32.8, *p* < 0.0001], and Dunnett’s *post hoc* testing revealed mice treated with 4% salt had significantly greater serum osmolarity than their controls in both the group (*p* < 0.0001) and single (*p* = 0.004) housing conditions (Figure 4D). Consistent with our earlier report (Mitchell et al., 2018), serum protein levels of Experiment 2 mice were elevated only in the 4% salt condition (data not shown).

### Correlations Between Behavior and Corticosterone Measures

**FIGURE 5:**
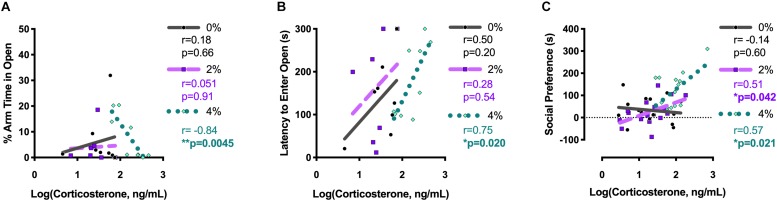
Pearson correlations between log-transformed corticosterone levels and behavior in the elevated plus maze and social interaction test. Pearson correlations between individual log-transformed serum corticosterone levels and percent arm time spent in the open arms of the elevated plus maze **(A)** and latency to first open arm entry in the elevated plus maze **(B)** were determined for group housed mice in Experiment 2. Pearson correlations between individual log-transformed serum corticosterone levels and social preference in the social interaction test were determined after collapsing across housing conditions for mice in Experiments 1 and 3 **(C)**. Pearson *r-*values are reported on the right side of each corresponding graph, along with the associated *p*-value. Bolded and asterisked *p*-values indicate values < 0.05.

Log-transformed corticosterone levels were graphed relative to individual behavior measures taken 18 h (Experiment 2) or 2 h (Experiments 1 and 3) prior to blood collection. The percent arm time spent in the open arms of the elevated plus maze was negatively correlated with corticosterone levels in 4% salt mice in Experiment 2 [Pearson *r* = −0.84; *F*(1,7) = 16.9, *p* = 0.0045; Figure 5A]. Likewise, latency to enter the open arms of the elevated plus maze was positively associated with corticosterone levels 18 h later in 4% salt mice [Pearson *r* = 0.75; *F*(1,7) = 9.1, *p* = 0.020; Figure 5B]. Neither 0% nor 2% salt mice had significant associations between corticosterone and these behavioral measures in either percent arm time in open arms [0%: Pearson *r* = 0.18; *F*(1,6) = 0.21, *p* = 0.66; 2%: Pearson *r* = 0.051; *F*(1,5) = 0.013, *p* = 0.91] or open arm latency [0%: Pearson *r* = 0.50; *F*(1,6) = 2.05, *p* = 0.20; 2%: Pearson *r* = 0.28; *F*(1,5) = 0.43, *p* = 0.54].

No significant correlations between log-transformed corticosterone and percent of marbles buried were detected for any treatment group in Experiment 2 [0%: Pearson *r* = −0.27; *F*(1,7) = 0.57, *p* = 0.48; 2%: Pearson *r* = 0.17; *F*(1,5) = 0.16, *p* = 0.71; 4%: Pearson *r* = −0.50; *F*(1,8) = 2.7, *p* = 0.14; data not shown].

Because no main effects of housing condition, and no interactions between housing and salt condition, were detected, social preference scores and log-transformed corticosterone values for mice in Experiments 1 and 3 were collapsed across housing condition before analyzing correlations (Figure 5C). Significant positive associations between corticosterone and social preference were found for both 2% [Pearson *r* = 0.51; *F*(1,14) = 5.0, *p* = 0.042] and 4% [Pearson *r* = 0.57; *F*(1,14) = 6.8, *p* = 0.021] salt conditions. No correlation was observed for mice in the 0% control condition [Pearson *r* = −0.14; *F*(1,14) = 0.28, *p* = 0.60].

## Discussion

Our observation that increased salt intake did not significantly affect anxiety-related behaviors, while contrary to our hypothesis, is intriguing given the pronounced reductions in compulsive nest building and enhanced social interaction exhibited by mice consuming the 4% salt solution. These lowered behavioral inhibitions, particularly in a mildly arousing social situation, may be indicative of an active coping response evoked by high salt intake, as we have previously observed in response to a highly arousing swim stress (Mitchell et al., 2018; Gilman et al., 2019a). Considering that nest building serves the purposes of affording environmental protection to mice and reducing their visibility to predators (Deacon, 2006), the reduced nest building exhibited by mice consuming high salt fits with this interpretation of attenuated behavioral inhibition. Consequently, the present study indicates that elevated salt consumption can substantially alter mildly stressful, ethologically relevant behaviors.

Previously, we reported that high salt intake elevates serum corticosterone levels in the absence of any behavior testing (Mitchell et al., 2018). For the present study, we evaluated corticosterone either 2 h after social interaction testing, or 18 h after elevated plus maze and marble burying tests. Corticosterone levels across the treatment conditions were similar to those from our previous report (Mitchell et al., 2018), suggesting that these behavior tests alone did not significantly affect corticosterone levels, and supporting our categorization of them as mildly stressful. Nonetheless, we observed positive associations between social interaction behavior and serum corticosterone levels 2 h later in animals consuming high salt, but not in controls. This is notable, particularly considering that social behavior in the 2% salt group was not significantly different from controls, nor were their serum Na^+^ or osmolarity levels elevated, despite their elevated drinking solution consumption (Mitchell et al., 2018). Failure to observe increased serum Na^+^ and osmolarity in the 2% salt group might reflect storage of excess Na^+^ in subcutaneous lymphatic vessels (Titze et al., 2005). Overall, the positive association between social behavior and elevated serum corticosterone is, nevertheless, consistent with our report of increased microglial activation in stress responsive-brain regions of mice consuming 2% salt, despite having no change in serum osmolarity (Gilman et al., 2019a and present findings). Whether behavioral and stress hormone effects observed in the present study involved proinflammatory signaling through activated microglia is not known, but this seems a reasonable possibility given that elevated salt intake has been reported to activate immune lymphocytes (Kleinewietfeld et al., 2013; Faraco et al., 2018), potentially promoting their extravasation into the brain (Li et al., 2019). Such a process could potentially underlie high salt intake-related microglial activation and modulation of neural circuits controlling stress-related behavior.

Moreover, corticosterone levels were selectively correlated with behavior in the elevated plus maze (but not marble burying test) in the 4% salt group, despite an absence of overt treatment group differences in this behavior test. This becomes even more remarkable considering the time gap of 18 h between behavior testing in the elevated plus maze and blood collection, versus the 2 h between social interaction testing and blood collection. Consequently, our findings suggest that stress-relevant physiological responses to mildly arousing stimuli (i.e., elevated plus maze and social interaction) may be prolonged under conditions of elevated salt intake. Alternatively, and consistent with our previous reports (Mitchell et al., 2018), salt consumption-related physiological shifts might stabilize under conditions of continued consumption (6 days), and thereby influence the degree of subsequently observed behavioral changes.

We did not observe an impact of single housing on behavioral changes elicited by salt consumption. This is not surprising, given that individually housed mice still experienced olfactory and auditory stimuli from neighboring cages in the housing room (Van Loo et al., 2003), and our mice had only been singly housed for 1 week (Võikar et al., 2005). The increased social engagement displayed by mice consuming 4% salt may be driven, at least in part, by activation of arginine vasopressin (AVP) neurons. We have reported increased AVP neuron activation in the paraventricular nucleus of the hypothalamus (PVN) in 4% salt-consuming mice (Mitchell et al., 2018). In agreement with this finding, AVP mRNA has been reported to increase in the PVN of mice consuming 2% salt in drinking water for 5 days (Ozaki et al., 2004). Social behaviors are strongly influenced by AVP (see review by Albers, 2012), so despite the association between social preference and corticosterone levels, AVP may also be contributing to this behavioral shift. This is consistent with the physiological stress of high salt intake activating corticotropin-releasing factor- and AVP-expressing hypothalamic neurons, which together drive HPA axis activity and hence corticosterone levels, as well as modulate downstream emotion-related behavioral circuits (Lucassen et al., 2014; Herman and Tasker, 2016).

We have observed that juvenile corticosterone levels in male mice following a social stress predicted voluntary social behavior 30 days later in early adulthood (Latsko et al., 2016). The present findings are the first, to our knowledge, to indicate a significant correlation between corticosterone levels and social interaction in the absence of any overt social stress (e.g., defeat by a dominant/resident rodent). However, Koolhaas and colleagues reported a significant positive correlation between blood pressure and corticosterone levels immediately after social defeat in rats (Fokkema et al., 1988). Corticosterone potentiates catecholamine-mediated vascular contraction (Fowler and Chou, 1961), and sympathetic nerve activity may be enhanced under conditions of persistent high sodium intake (Toney et al., 2003; Oh et al., 2016; Matthews et al., 2017). Non-canonical effects of elevated corticosterone, such as impaired clearance of serotonin and the behavioral sequelae thereof, should also be considered (Baganz et al., 2010).

This information, combined with the present study’s findings, suggests corticosterone-mediated behavioral changes resulting from elevated salt intake might feed forward to promote maladaptive stress responses, both cardiovascular and behavioral. Adding in that appetite for salt can be enhanced by adrenal hormones (Shelat et al., 1999; Daniels and Fluharty, 2004), some organisms may encounter (perceived) stressful circumstances that prime them for a downward cardiovascular and behavioral spiral when high sodium access is unrestricted. Thus, salt consumption may indeed be a key contributing link between cardiovascular and stress-related neuropsychiatric diseases (Player and Peterson, 2011; Cohen et al., 2015; Seldenrijk et al., 2015; Tully et al., 2016; Edmondson and Känel, 2017), with behavioral disruptions potentially emerging before detectable cardiovascular shifts.

Future work should focus on assessing the influence of more prolonged salt consumption (>1 week) on behavioral disruptions relevant to stress-related neuropsychiatric disorders, concurrent with longitudinal cardiovascular measures such as blood pressure. Given evidence that both sexes share many risk factors for cardiovascular disease (Appelman et al., 2015), study of females will be an important avenue for future investigations. Exploring whether adrenalectomy or glucocorticoid receptor antagonism negates the elevated social behavior observed in mice consuming 4% salt would address the lingering question about any causative behavioral contributions of adrenal steroids such as corticosterone. However, interpretations could be partially confounded by the necessity to supply salt in the drinking water of controls for this surgical manipulation, though it is relatively small (0.45%) compared to salt concentrations used here. Further investigations into how specific pro-inflammatory cytokines contribute to salt-induced neuroinflammation and behavioral inhibition, or which AVP circuits are activated, would shed much-needed light on how salt consumption contributes to stress-related psychopathology. In short, there remain many avenues for investigating the importance of salt consumption on stress responsivity to better understand mental and cardiovascular health as well as their possible comorbid pathogenesis.

## Data Availability Statement

The datasets generated for this study are available on request to the corresponding author.

## Ethics Statement

All experiments were approved by the Institutional Animal Care and Use Committee at the University of Texas Health Science Center at San Antonio, and complied with the National Research Council’s Guide for the Care and Use of Laboratory Animals, 8th Ed.

## Author Contributions

TG, GT, and LD conceptualized experiments. TG, CG, and MA executed experiments. TG, MA, NM, and GT statistically analyzed and graphed data. TG, CG, NM, GT, and LD interpreted results. TG wrote initial manuscript draft. All authors critically read and revised manuscript, and approved submitted version.

## Conflict of Interest

The authors declare that the research was conducted in the absence of any commercial or financial relationships that could be construed as a potential conflict of interest.
